# Single-use digital flexible cystoscope for double J removal versus reusable instruments: a prospective, comparative study of functionality, risk of infection, and costs

**DOI:** 10.1007/s00345-023-04636-0

**Published:** 2023-10-02

**Authors:** Marco Oderda, Anastasios Asimakopoulos, Valerio Batetta, Andrea Bosio, Ettore Dalmasso, Ivano Morra, Eugenia Vercelli, Paolo Gontero

**Affiliations:** 1 Division of Urology, Department of Surgical Sciences, Molinette Hospital, University of Turin, Turin, Italy; 2 Division of Urology, Tor Vergata, Rome, Italy; 3 Division of Urology, Santa Croce e Carle Hospital, Cuneo, Italy

**Keywords:** Isiris, Flexible cystoscope, Functionality, Urinary tract infections, Cost

## Abstract

**Background:**

The removal of ureteral stent can be performed with disposable or reusable flexible cystoscopes, but limited comparative data are available on functionality, risk of infections, and costs.

**Methods:**

We performed a multicentric, prospective, observational study on patients undergoing in-office ureteral stent removal with Isiris-α^®^ or a reusable Storz™ flexible cystoscope. Study endpoints were the functionality and effectiveness of the devices, the rate of postoperative bacteriuria and UTIs, and the costs of the procedure.

**Results:**

A total of 135 patients were included, 80 (59.2%) treated with reusable cystoscopes and 55 (40.8%) with Isiris-α^®^. No significant baseline differences between groups were detected. Isiris-α^®^ outperformed the reusable device in terms of quality of vision (*p* 0.001), manoeuvrability (*p* 0.001), grasper functionality (*p* < 0.001), and quality of the procedure (*p* 0.01). Mean procedure time was shorter with Isiris-α^®^ (*p* < 0.001) due to a shorter instrument preparation time (*p* < 0.001). No differences were found in terms of perceived patient pain (*p* 0.34), nor postoperative bacteriuria or symptomatic UTIs. According to our cost analysis, the in-office procedure performed with Isiris-α^®^ was more expensive (+ 137.8€) but was independent from instrument turnover or disinfection. Among limitations of study we acknowledge the lack of randomization, the use of antibiotic prophylaxis in several patients, and the high rate of missing preoperative urine cultures.

**Conclusions:**

Isiris-α^®^ outperforms reusable cystoscopes for in-office ureteral stent removal in terms of total operative time and quality of the procedure, at the cost of being more expensive. No significant differences in postoperative bacteriuria or symptomatic UTIs were found.

**Supplementary Information:**

The online version contains supplementary material available at 10.1007/s00345-023-04636-0.

## Introduction

Isiris-α^®^ (Coloplast, Denmark) is an effective grasper-integrated disposable device dedicated to JJ removal. It is now a viable alternative to the standard reusable flexible cystoscope, with the potential benefit to streamline the process of stent removal [1, 2]. A recent randomized controlled trial showed that disposable cystoscopes are comparable to reusable ones in terms of pain score and surgeons’ and patients’ satisfaction, with an advantage in cost-effectiveness [3]. However, it is difficult to give an accurate and generalized estimation of the procedural costs given the different settings and reimbursement of JJ removal in each institution. In a multicentric evaluation of reported costs of JJ removal, the use of Isiris-α^®^ has been shown to bring significant improvement in organization and turnover, which finally impacts on the cost-effectiveness of the procedure [2]. Furthermore, reusable channelled cystoscopes need high-level disinfection (HLD) process or low-temperature sterilization, with risks of inadequate processing, residual contaminants, or scope damages [4]. It has been reported that the risk of infection outbreaks through endoscopes occurs even when the guidelines for disinfection are correctly followed [5].

The aim of the present study was to prospectively compare Isiris-α^®^ with a reusable flexible cystoscope in terms of functionality, rate of postoperative urinary tract infections (UTIs), and costs.

## Patients and methods

We performed a prospective, comparative, non-randomized single-blinded study enrolling 135 patients undergoing JJ removal, irrespectively of their condition, in three Italian institutions from June 2022 to March 2023. These patients were divided in two groups: Isiris-α^®^ (*N* = 55) and reusable cystoscope (*N* = 80). Randomization could not be performed because cystoscope type selection depended on its availability in each institution. This study was approved by the “A.O.U. Città della Salute e della Scienza” ethics committee as coordinator centre (N. 00515/2020).

### Cystoscopy procedure

**Fig. 1:**
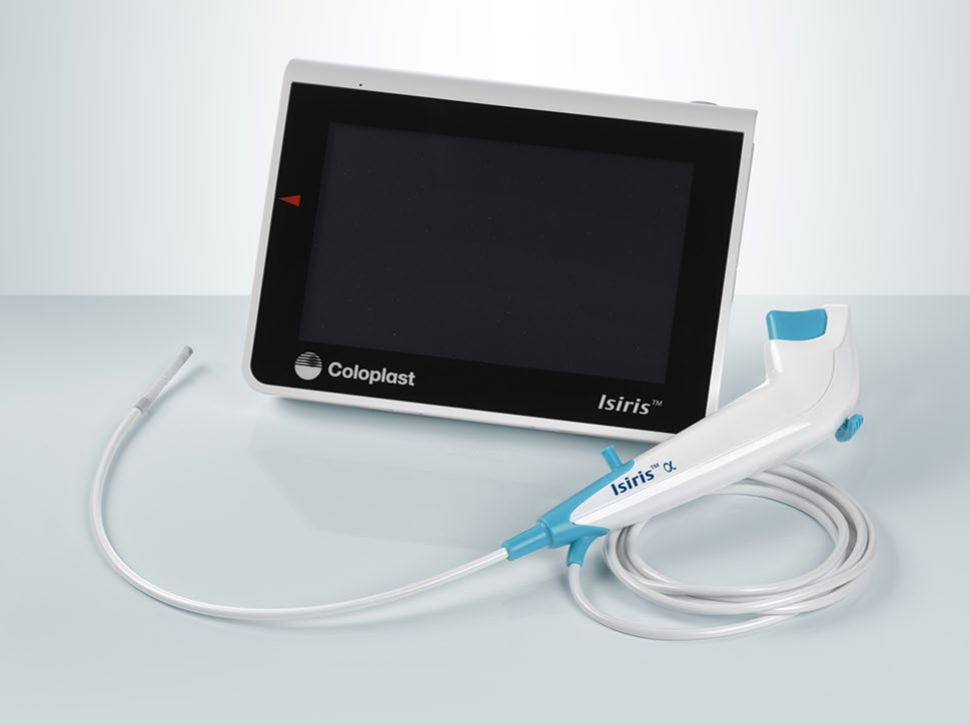
Isiris-α^®^

The procedures were performed by residents (under the supervision of senior urologists) either with Isiris-α^®^ (Fig. 1) or with the reusable Storz™ flexible cystoscope with the use of an external grasper. An intra-urethral 2% lidocaine gel injection was done just before the procedure.

### Outcomes measurements and endpoints

Baseline data included gender, age, type and duration of indwelling ureteral stents, and ureteral stent indication. Intraoperative data included cystoscope preparation duration, stent removal duration (time from cystoscope insertion to stent removal), total procedural time, and damage to the cystoscope or the grasper. Surgeons’ opinions on quality of vision, manoeuvrability, grasper functionality, and overall quality of the procedure were collected after the procedure: a questionnaire specifically developed to evaluate these items was filled after each procedure. Patients were asked about their pain score using the 10-point visual analogue scale (VAS). Patients were asked for urine culture within the previous 7 days before and 7 days after the procedure, and they were followed up for 30 days to rule out postoperative UTIs. Data on pre- or postoperative antibiotic treatment or prophylaxis were collected. As for the costs of the procedure, we considered the following: cost of the single-use device Isiris™; cost of Storz^®^ flexible cystoscope and the grasper(s), to be divided by the number of procedures performed with a single device; cost of maintenance and repair(s) of the reusable device; cost of the urologist work and further personnel (i.e. nurse); cost of cleaning / rinsing (detergent); and cost of HLD.

Primary endpoint of study was the evaluation of the functionality of Isiris-α^®^ versus the reusable flexible cystoscopes Storz^®^ for JJ removal. Secondary endpoints were the evaluation of the rate of postoperative UTIs and the costs of the procedure.

### Statistical analyses

Statistical analyses were performed with SPSS version 28.0 (IBM Corp, Armonk, NY, USA). Quantitative data are shown as median and interquartile range (IQR) and were compared using median test and Mann–Whitney test, while qualitative data are shown as frequencies and percentages and were compared using Pearson’s chi-squared test. Missing data were treated with pairwise deletion. Statistical significance was considered at 2-sided *p* < 0.05.

## Results

Baseline characteristics of our patients are detailed in Table 1. There were no statistically significant differences between the two groups in terms of age, gender, type of ureteral stents, nor stent duration.

**Table 1:** Baseline characteristics

Characteristic	Overall	Isiris	Reusable cystoscope	*p*
*Number of patients*	135	55	80	–
*Gender, n (%)*				0.71
Male	86 (63.7%)	34 (61.8%)	52 (65%)	
Female	49 (36.3%)	21 (38.2%)	28 (35%)	
*Age, median, years (IQR)*	61 (17)	60 (15)	61 (19)	0.82
*Ureteral stents, n (%)*				0.08
Double J	129 (95.6%)	55 (100%)	74 (92.5%)	
J-fil	6 (4.4%)	0 (0%)	6 (7.5%)	
*Ureteral stent indication, n (%)*				0.16
Hydronephrosis	9 (6.7%)	3 (5.5%)	6 (7.5%)	
Ureteroscopy	88 (65.2%)	41 (74.5%)	47 (58.8%)	
Kidney transplant	25 (18.5%)	9 (16.4%)	16 (20.0%)	
Surgery (pyeloplasty, ureteral reimplantation)	13 (9.6%)	2 (3.6%)	11 (13.8%)	
*Stent duration, median, days (IQR)*	27 (14.5)	23 (13.5)	30 (20)	0.09

No statistically significant differences were noted in terms of preoperative and postoperative positive urine cultures between the two groups (5 and 4 in the Isiris-α^®^ group, 1 and 2 in the reusable cystoscopes group, respectively). However, these results were affected by missing data, as 40% and 30% of our patients did not perform urine cultures in the preoperative and postoperative setting, respectively. A high rate of patients received prophylactic or therapeutic antibiotics in the preoperative setting (34% in the Isiris-α^®^ group and 33% in the reusable cystoscopes group), whereas only 3% and 5% of them needed antibiotics postoperatively. No statistically significant differences were found in terms of post-procedural symptomatic UTIs, recorded in only 1 case in the Isiris-α^®^ group and 4 in the reusable cystoscopes group (*p* 0.36). Findings about urine cultures and UTIs are shown in Supplementary Fig. 1.

Table 2 resumes the characteristics of the procedure. Isiris-α^®^ outperformed the reusable device in terms of quality of vision (judged “very good” in 94.5% vs 58.8% of cases, *p* 0.001), manoeuvrability (judged “very good” in 92.7% vs 62.5% of cases, *p* 0.001), grasper functionality (judged “easy” in 92.7% vs 62.5% of cases *p* < 0.001), and overall quality of the procedure (judged “very good” in 83.6% vs 55.0% of cases, *p* 0.01). Mean total procedure time was shorter with Isiris™ (8.7 vs 12.4 min, *p* < 0.001) due to a shorter instrument preparation time (4.1 vs 7.5 min, *p* < 0.001). No differences were found in terms of perceived patient pain (median VAS score 3 in both groups, *p* 0.34). No damages to the cystoscopes were noted in our series, whereas 4 reusable graspers needed to be replaced because of unrepairable damage.

**Table 2:** Characteristics of the procedure

Characteristic	Overall	Isiris	Reusable cystoscope	*p*
*Quality of vision*				**< 0.001**
Bad	1 (0.7%)	0 (0.0%)	1 (1.3%)	
Poor	8 (5.9%)	1 (1.8%)	7 (8.8%)	
Fair	6 (4.4%)	0 (0.0%)	6 (7.5%)	
Good	21 (15.6%)	2 (3.6%)	19 (23.8%)	
Very good	99 (73.3%)	52 (94.5%)	47 (58.8%)	
*Manoeuvrability*				**0.001**
Bad	2 (1.5%)	0 (0.0%)	2 (2.5%)	
Poor	6 (4.4%)	2 (3.6%)	4 (5.0%)	
Fair	9 (6.7%)	2 (3.6%)	7 (8.8%)	
Good	17 (12.6%)	0 (0.0%)	17 (21.3%)	
Very good	101 (74.8%)	51 (92.7%)	50 (62.5%)	
*Grasper functionality*				**< 0.001**
Easy	102 (75.6%)	51 (92.7%)	51 (63.7%)	
Normal	21 (15.6%)	4 (7.3%)	17 (21.3%)	
Difficult	12 (8.9%)	0 (0.0%)	12 (15.0%)	
*Cystoscope preparation duration, minutes, mean (SD)*	6.1 (3.7)	4.1 (2.2)	7.5 (3.9)	**< 0.001**
*Cystoscope preparation duration, minutes, median (IQR)*	5 (3)	5 (4)	7 (5)	**< 0.001**
*Stent removal duration, minutes, mean (SD)*	4.8 (2.9)	4.6 (2.8)	5.0 (2.9)	0.38
*Stent removal duration, minutes, median (IQR)*	5 (2)	5 (4)	4 (2)	0.89
*Total procedural time, minutes, mean (SD)*	10.9 (5.7)	8.7 (4.8)	12.4 (5.8)	**< 0.001**
*Total procedural time, minutes, median (IQR)*	10 (6)	10 (7)	10 (7)	**0.01**
*Cystoscope damage, n (%)*	0 (0%)	0 (0%)	0 (0%)	–
*Grasper damage, n (%)*	4 (3.0%)	0 (0.0%)	4 (5.0%)	0.14
*Quality of procedure, n (%)*				**0.01**
Bad	1 (0.7%)	0 (0.0%)	1 (1.3%)	
Poor	5 (3.7%)	1 (1.8%)	4 (5.0%)	
Fair	11 (8.1%)	3 (5.5%)	8 (10.0%)	
Good	28 (20.7%)	5 (9.1%)	23 (28.7%)	
Very good	90 (66.7%)	46 (83.6%)	44 (55.0%)	
*Pain, VAS score, median (IQR)*	3 (4)	3 (4)	3 (5)	0.12

According to our cost analysis, the in-office procedure performed with Isiris-α^®^ cost 250€ for the purchase of the instrument, plus 39.2€ for the urologist/nurse work. The same procedure performed with a reusable cystoscope cost 44.1€ for the purchase of the instrument/grasper (estimated on the total number of procedures performed during the lifespan of the instrument), plus 14.1€ for the estimated repairs, plus 17.5€ for HLD. We had to add the cost of the purchase of 4 new graspers during the period of study (36.5€ per procedure). Overall, Isiris-α^®^ was more expensive (+ 137.8€ per procedure) but was independent of the instrument turnover or disinfection.

## Discussion

In the last years, disposable flexible cystoscopes have become a strong presence in the market of cystoscopes. Among them there is the grasper-integrated digital flexible cystoscope Isiris-α^®^ that is dedicated to JJ removal. Isiris-α^®^ has several advantages as compared to the reusable instruments: it allows the JJ removal in-office without the aid of any assistant thanks to the possibility of activating the grasper by pushing a button on the handle of the device [6]. Furthermore, it does not require decontamination nor sterilization, which might impact on the longevity of reusable instruments [7]. Reusable cystoscopes have been identified by the FDA among the medical devices that pose a greater likelihood of microbial transmission and represent a high risk of infection (subclinical or clinical) if they are not adequately reprocessed. Some risk of infection outbreaks through channelled endoscopes remains even when the guidelines for disinfection are correctly followed [5]. Finally, Isiris-α^®^ is independent of the turnover of reusable instruments, allowing potentially a higher number of procedures per session.

Recently, several trials have been published to compare the performances of disposable and reusable cystoscopes. In 2022, Alkhamees et al. conducted a randomized controlled trial in Saudi Arabia on 128 patients who underwent JJ removal with Isiris-α^®^ or reusable Olympus cystoscopes. They showed that both disposable and reusable cystoscopes are comparable in terms of pain score and surgeons’ and patients’ satisfaction. Isiris-α^®^ resulted to be more cost effective in their health system, considering that all procedures in their study were conducted in the operatory room and not in an outpatient setting [3]. The same comparison was performed by Navarrete et al. in 2023 in a non-randomized prospective study, concluding that Isiris-α^®^ is comparable to the reusable cystoscope in terms of pain and endoscopy time [8]. In 2023, Holmes et al. conducted a non-inferiority randomized controlled trial comparing the disposable Ambu^®^ aScope™ 4 Cysto System with the reusable Olympus CYF-VH cystoscopes, concluding that both instruments are comparable in terms of procedure completion, light quality, image quality, and manoeuvrability [9]. Of note, the disposable instruments by Ambu^®^ differ from Isiris-α^®^ for the lack of the integrated grasper.

All the aforementioned comparative studies confirmed the effectiveness and functionality of the disposable instruments, even though no superiority was demonstrated. On the other hand, in the present study we showed that Isiris-α^®^ clearly outperformed the reusable Storz^®^ device in terms of quality of vision, manoeuvrability, grasper functionality, and overall quality of the procedure. Our procedures were shorter with the use of Isiris-α^®^, mainly for the shorter instrument preparation time. The difference between our study and the others might be related to an inferior quality of our reusable devices, or to the fact that in our study the procedures were performed by residents, more prone to new technologies. It is undeniable that sometimes the external graspers do not properly work when the cystoscope has a high degree of deflection, issue that is overcome with the grasper-integrated devices. In line with Alkhamees et al. [3] and Navarrete et al. [8], we did not find any differences in terms of perceived patient pain. Furthermore, in our series the duration of indwelling stents was shorter in the Isiris-α^®^, even though the difference did not reach statistical significance. It has been shown that single-use cystoscopes can lead to shorter JJ indwell duration, being independent from instruments’ turnover and disinfection, and allowing the performance of a higher number of procedures per session [2, 6].

An interesting alternative for JJ removal is represented by magnetic stents that can be removed by a special catheter-like retrieval instrument with a magnetic tip [10]. A recent randomized study on 60 patients concluded that removal of magnetic stents with a dedicated retriever under ultrasound guidance represents a less time-consuming and more comfortable way to remove ureteral stents, avoiding the need for cystoscopy [11]. The advantages of magnetic stents in terms of shorter removal time, less pain during removal, and low cost in comparison to conventional stents were confirmed in a systematic review that included seven studies [12]. However, we must consider that blinded magnetic stent removal might be difficult or even dangerous in not-ordinary cases such as incrusted stents, patients with big prostates, or kidney transplant patients. Another viable alternative for the in-office removal of ureteral stents in females is represented by rigid cystoscopes that are still used in several institutions. We believe that this option is surely quick and cost effective, but still more bothersome than using flexible instruments. Interestingly, in 2009 a randomized controlled trial comparing the tolerability of rigid versus flexible cystoscopy in women concluded that discomfort during and after the procedure is minimal in both the groups [13]. Nevertheless, a prospective analysis on 1.320 patients undergoing diagnostic cystoscopy concluded that flexible instruments are associated with a lower pain level in both men and women and should be used for both genders [14].

During our study period, no damages to the reusable cystoscopes were recorded, but four of our reusable graspers needed to be replaced because of unrepairable damage. The need for periodic replacements of the grasper needs to be considered in the cost analysis, as well as the potential increase in the number of procedures performed per session with the use of Isiris-α^®^. Furthermore, procedures performed with reusable instruments obviously need an endoscopic column with a monitor and a light source. Considering only the estimated costs per procedure, however, in our institution Isiris-α^®^ was more expensive than the reusable instruments (+ 137.8€ per procedure).

As for the risk of infection, in our series one case of symptomatic UTI was reported in the Isiris-α^®^ group, while four cases were observed in the reusable cystoscope group. In the literature, the incidence of bacteriuria and febrile UTI after cystoscopy ranges from 1.9 to 9% [3, 15]. Unfortunately, in our study we could not adequately evaluate the incidence of pre- and postoperative bacteriuria, because many patients were not compliant about the performance of urine cultures. This evaluation was also affected by the non-negligible rate of antibiotics taken in the preoperative setting, over 30% in both groups, reflecting a tendency to antibiotic over-treatment but also the 20% rate of kidney transplant patients enrolled in our series. In those patients, complications and infections must be avoided at any cost.

A final comment involves the environmental impact of single-use devices as compared to the life cycle of reusable instruments. A recent study compared the carbon footprint of single-use versus reusable flexible cystoscopes based on waste production and estimated carbon emissions produced by disposal, manufacture, and cleaning. Interestingly, according to their results disposable flexible cystoscopes had a significantly lower impact on the environment in terms of carbon footprint and landfill, highlighting the sustainability of these devices [16].

Among the main limitations of study, we acknowledge the lack of randomization, the use of antibiotic prophylaxis in several patients, and the high rate of missing preoperative urine cultures.

## Conclusions

In our study, Isiris-α^®^ outperformed reusable cystoscopes for in-office ureteral stent removal in terms of total operative time, quality of vision, manoeuvrability, grasper functionality, and overall quality of the procedure, at the cost of being more expensive. No significant differences in postoperative bacteriuria or symptomatic UTIs were found, considering the limited sample size.

### Supplementary Information

Below is the link to the electronic supplementary material.

Supplementary Fig. 1 A. Positive urine cultures rates. B. Antibiotic treatment rates. C. Symptomatic UTIs rates (TIF 143 KB)

## Data Availability

The data that support the findings of this study are available from the corresponding author, M.O., upon reasonable request.
