# Supplementary figures and images for: Single-use digital flexible cystoscope for double J removal versus reusable instruments: a prospective, comparative study of functionality, risk of infection, and costs
Source: World J Urol. 2023 Oct 2;41(11):3175–80. doi: 10.1007/s00345-023-04636-0 (PMC10632259; doi:10.1007/s00345-023-04636-0)

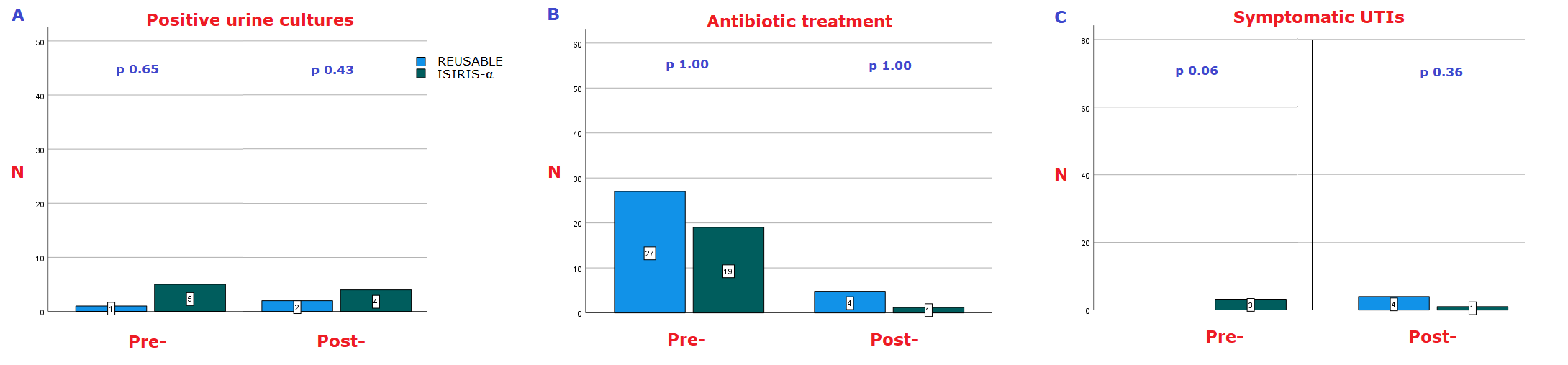

Supplement: Supplementary file 1 — Supplementary Fig. 1 A. Positive urine cultures rates. B. Antibiotic treatment rates. C. Symptomatic UTIs rates (TIF 143 KB) [file 345_2023_4636_MOESM1_ESM.tif]
